# Sustainable neonatal CLABSI surveillance: consensus towards new criteria in the Netherlands

**DOI:** 10.1186/s13756-021-00900-3

**Published:** 2021-02-05

**Authors:** I. E. Heijting, T. A. J. Antonius, A. Tostmann, W. P. de Boode, M. Hogeveen, J. Hopman, the Working Group on Neonatal Infectious Diseases of the Section of Neonatology of the Dutch Paediatric Society, R. F. Kornelisse, E. J. d’ Haens, M. A. C. Hemels, J. U. M. Termote, V. Bekker, S. J. Jansen, D. H. Visser, J. P. F. van der Sluijs-Bens, M. C. Hutten, K. A. Bergman

**Affiliations:** 1 Department of Paediatrics, Division of Neonatology, Amalia Children’s Hospital, Radboud University Medical Center, Radboud Institute for Health Sciences, Internal Postal Code 804, Geert Grooteplein Zuid 10, 6525GA Nijmegen, The Netherlands; 2 Unit of Hygiene and Infection Control, Department of Medical Microbiology, Radboudumc Center for Infectious Diseases, Radboud University Medical Center, Nijmegen, The Netherlands; 3 Department of Quality and Safety, Department of Medical Microbiology, Radboudumc Center for Infectious Diseases, Radboud University Medical Center, Nijmegen, The Netherlands

**Keywords:** Central line-associated bloodstream infections (CLABSI), Catheter-related infections, Cross infection, Hospital-acquired infections (HAI), Neonatal intensive care unit (NICU), Newborn, Infection control, Epidemiological monitoring, Quality improvement, Surveillance

## Abstract

**Background:**

Central line-associated bloodstream infections (CLABSI) are a main focus of infection prevention and control initiatives in neonatal care. Standardised surveillance of neonatal CLABSI enables intra- and interfacility comparisons which can contribute to quality improvement. To date, there is no national registration system for CLABSI in neonatal care in the Netherlands and several criteria are used for local monitoring of CLABSI incidence rates. To achieve standardised CLABSI surveillance we conducted a consensus procedure with regard to nationwide neonatal CLABSI surveillance criteria (SC).

**Methods:**

A modified Delphi consensus procedure for the development of nationwide neonatal CLABSI SC was performed between January 2016 and January 2017 in the Netherlands. An expert panel was formed by members of the Working Group on Neonatal Infectious Diseases of the Section of Neonatology of the Dutch Paediatric Society. The consensus procedure consisted of three expert panel rounds.

**Results:**

The expert panel achieved consensus on Dutch neonatal CLABSI SC. Neonatal CLABSI is defined as a bloodstream infection occurring more than 72 h after birth, associated with an indwelling central venous or arterial line and laboratory confirmed by one or more blood cultures. In addition, the blood culture finding should not be related to an infection at another site and one of the following criteria can be applied: 1. a bacterial or fungal pathogen is identified from one or more blood cultures; 2. the patient has clinical symptoms of sepsis and 2A) a common commensal is identified in two separate blood cultures or 2B) a common commensal is identified by one blood culture and C-reactive protein level is above 10 mg/L in the first 36 h following blood culture collection.

**Conclusions:**

The newly developed Dutch neonatal CLABSI SC are concise, specified to the neonatal population and comply with a single blood culture policy in actual neonatal clinical practice. International agreement upon neonatal CLABSI SC is needed to identify best practices for infection prevention and control.

## Background

Neonatal central line-associated bloodstream infections (CLABSI) are associated with significant morbidity and mortality and burden the healthcare system with substantial costs [1]. These largely preventable hospital-acquired infections (HAI) are a main focus of infection prevention and control initiatives and are considered to be an important quality indicator in neonatal care [2–4]. Surveillance of CLABSI is essential to evaluate the success of preventive interventions and to measure trends over time [5–7]. Standardised CLABSI monitoring enables intra- and interfacility incidence rate comparisons which contributes to practice improvement [7, 8]. National registration systems of CLABSI have shown to be beneficial in this matter [9].

To date, there is no national registration system for CLABSI in neonatal care in the Netherlands. Across Dutch neonatal intensive care units (NICUs), several different sets of CLABSI criteria and surveillance methods are used for local monitoring of CLABSI incidence rates resulting in considerable variation in incidence numbers impeding accurate interfacility comparison [10]. Furthermore, in some Dutch NICUs, CLABSI surveillance is not routinely performed. Different CLABSI criteria are available in the literature and research case definitions vary from surveillance definitions. In addition, case definitions suitable for CLABSI surveillance (often retrospective) differ from definitions used for clinical diagnosis. Elements included in existing surveillance criteria (SC) range from simple definitions based on mainly laboratory data to detailed clinical CLABSI definitions specified to the neonatal population [11]. Case definitions for infection surveillance established by the US National Healthcare Surveillance Network (NHSN) in partnership with the Centers for Disease Control and Prevention (CDC) have been adopted in whole or modified forms by numerous reporting agencies internationally [7]. However, the CDC CLABSI criteria are available for patients ≤ 1 year of age and are not specifically developed for use in newborns with a lower body weight and corresponding lower circulating blood volumes [12]. Likewise, the European Centre for Disease Control and Prevention (ECDC) adopted the CDC CLABSI criteria in modified form but without any specifications for different patient age groups [13]. CDC and ECDC criteria can potentially lead to underestimation of the neonatal CLABSI incidence, as Coagulase-negative Staphylococci sepsis (CoNS) requires one of four defined clinical symptoms and two positive cultures of blood specimen obtained on the same or consecutive calendar day [14]. However, due to low circulating volumes in newborns a single blood culture policy is applied in the Netherlands and confirmation of sepsis by a second blood specimen is often not performed. To adapt CLABSI criteria to the neonatal setting and to encourage surveillance support of neonatologists, the NEO-Krankenhaus Infektions Surveillance System (KISS) criteria were developed in Germany. The NEO-KISS surveillance system includes very low birthweight  (VLBW) infants (birthweight < 1500 g) and categorises CLABSI events as clinical sepsis (infection without a detected pathogen), laboratory-confirmed bloodstream infection with a detected pathogen (but not common commensal microflora) and laboratory-confirmed bloodstream infection with a common commensal as the sole pathogen [15]. Moreover, for case ascertainment, all NEO-KISS CLABSI categories require two or more of 16 clinical findings to be present. Using detailed clinical data in case definitions requires close involvement of clinicians which may result in time consuming and labour intensive surveillance methods based on manual data extraction [16]. Subjectivity in interpretation of clinical findings reduces interrater agreement and increases variability in incidence rates [17].

Our aim was to achieve standardised nationwide neonatal CLABSI surveillance. As a first step, we strived for national consensus with regard to the neonatal CLABSI SC. To increase validity and sustainability of neonatal CLABSI surveillance, the definitions had to be concise, suitable for the neonatal population and for use in the Dutch neonatal care setting. Here we report on the consensus procedure and present the newly developed neonatal CLABSI SC that are currently used in the Netherlands.

## Methods

### Design and setting

The Netherlands is a country with 17.4 million inhabitants and approximately 170,000 liveborn births per year [18]. There are nine level III-IV NICUs across seven university hospitals and two general hospitals. Together, they provide 195 NICU beds and facilitate on average 5000 NICU admissions each year [19].

A structured consensus procedure (modified Delphi method) for developing nationwide neonatal CLABSI SC in the Netherlands was performed between January 2016 and January 2017 [20].

### Participants

An expert panel was formed by the Working Group on Neonatal Infectious Diseases of the Section of Neonatology of the Dutch Paediatric Society. Clinical guidelines created by this working group are distributed and implemented nationally. The expert panel was compiled by representatives of eight of the nine NICUs in the Netherlands and consisted of twelve participants, ten of whom were neonatologists, one a paediatric resident and one a research nurse. Depending on the topic being discussed a microbiologist, specialist in paediatric infectious diseases, or other experts were being consulted. Two members of the expert panel were appointed as coordinators (IH, TA) of the consensus procedure.

### Data collection

**Fig. 1:**
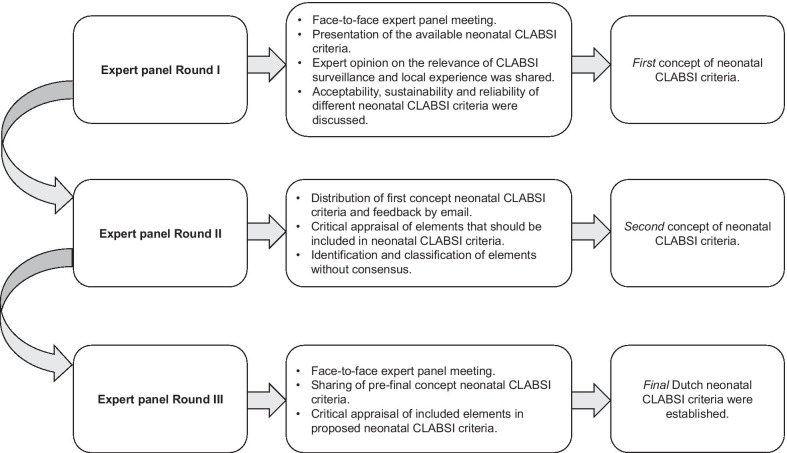
Detailed description and results of the structured consensus procedure

The modified Delphi consensus procedure consisted of three Expert Panel Rounds. Proceeding Expert Panel *Round I*, the two coordinators identified elements of case definitions for neonatal CLABSI surveillance by reviewing available CLABSI SC in literature and guidelines (Table 1). *Round I and III* consisted of a face-to-face expert panel meeting. In *Round II,* the first concept version of neonatal CLABSI SC was electronically distributed to the expert panel for feedback. Figure 1 shows a detailed description of the consensus procedure.

**Table 1:** The main features of currently used surveillance criteria of neonatal CLABSI summarised

	CDC	NEO-KISS	Dutch neonatal CLABSI criteria
Target patient population	≤ 1 year	Very low birthweight infants: birthweight < 1500 g	Neonates: postnatal age ≤ 28 days for term and up to postmenstrual age of 44 weeks for preterm infants
Description of CLABSI criteria	Laboratory-confirmed bloodstream infection (1) with a detected pathogen OR (2) with the same common commensal^a^ confirmed by a second blood specimen	Laboratory-confirmed bloodstream infection (1) with a detected pathogen OR (2) with CoNS confirmed by a second blood specimen or one out of 4 laboratory elements	Laboratory-confirmed bloodstream infection (1) with a detected pathogen OR (2) with same common commensal^a^ confirmed by a second blood specimen OR (3) with a common commensal and CRP > 10 mg/L
		Clinical sepsis (3) no detected pathogen	
Clinical findings used in criteria	One out of four^b^ clinical symptoms for CoNS CLABSI criteria	Two or more out of 16^c^ findings bundled in seven categories for all CLABSI criteria	Clinical symptoms of neonatal sepsis according to the treating physician for “common commensal CLABSI criteria”
Challenges for application in the Dutch setting	Two blood cultures for one event (not common practice, single blood culture policy)	Numerous clinical elements (labour intensive surveillance and possible interference with interrater agreement)	–

CDC, Centers for Disease Control; CLABSI, central line-associated bloodstream infections; CoNS, Coagulase-negative Staphylococci species; CRP, C-reactive protein; NEO-KISS, NEO-Krankenhaus Infektions Surveillance System

^a^According to the NHSN Master Organism List

^b^CDC clinical findings: fever (> 38 °C), hypothermia (< 36.5 °C), apnoea, or bradycardia

^c^NEO-KISS clinical findings: (1) fever (> 38 °C) or temperature instability or hypothermia (< 36.5 °C); (2) tachycardia (> 200/min) or new increasing bradycardia (< 80/min); 3) capillary refill time > 2 s; (4) new or increasing apnoea (> 20 s); (5) otherwise unexplained metabolic acidosis (BE < − 10 mval/L); (6) new onset of hyperglycaemia (> 140 mg/dL); (7) other sighs of sepsis (skin colour, biochemical signs, increasing oxygen requirement, unstable general status, apathy)

## Results

### Consensus procedure

Following Expert Panel *Round I,* the first concept for Dutch neonatal CLABSI SC was developed. A subsequent iterative process of consultation with the expert panel in *Round II* and revision of the concept resulted in consensus concerning Dutch neonatal CLABSI SC in *Round III*. Table 1 summarizes the main features of the currently used SC of neonatal CLABSI. The results of the structured consensus procedure are outlined in Fig. 1.

### Nationwide neonatal CLABSI surveillance criteria

**Fig. 2:**
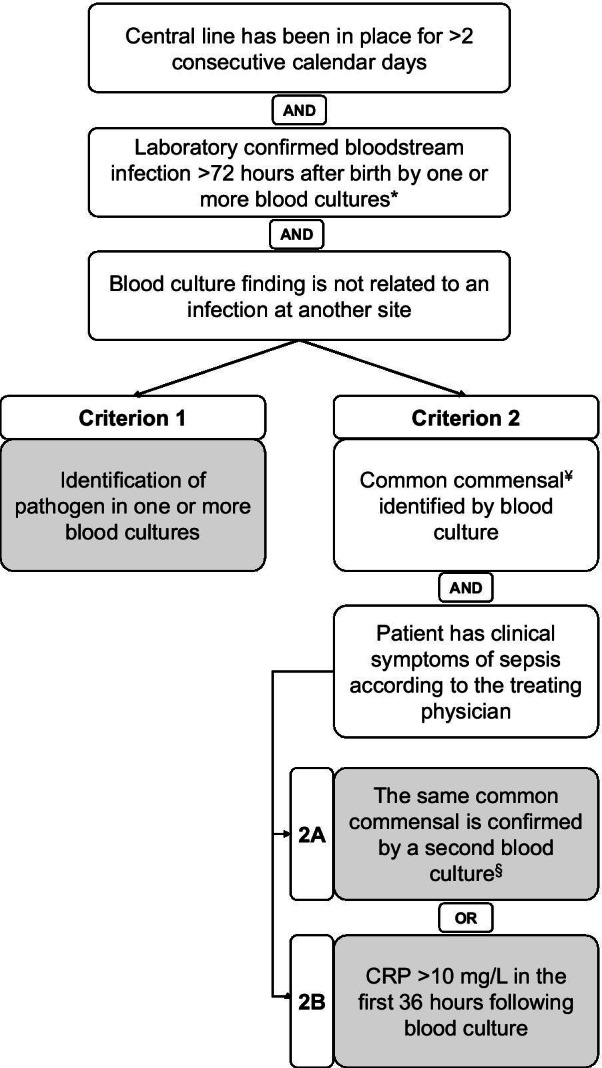
Summary of the Dutch neonatal CLABSI surveillance criteria. *Blood specimens for blood culture can be collected through peripheral venepuncture or can be sampled through central lines and should be obtained in compliance with existing guidelines before the start of antibiotic treatment following hygienic precautions. ^¥^Common commensals according to NHSN Master Organism List include, but are not limited to, Coagulase-negative Staphylococci (CoNS). ^§^Confirmation by a second blood specimen means two or more blood specimens are sampled on separate occasions. These separate occasions are defined as at least two separate blood samples collected on the same or consecutive calendar days

The outcome of the consensus procedure was the establishment of Dutch neonatal CLABSI SC which are summarized in Fig. 2. Neonatal CLABSI is defined as the occurrence of a laboratory confirmed bloodstream infection occurring more than 72 h after birth that was associated with an indwelling central line. The definition used for ‘central line’ was adopted from the CDC: “A *central line* is defined as an arterial or venous intravascular catheter that terminates at or close to the heart or in one of the great vessels, and is used for infusion, withdrawal of blood or hemodynamic monitoring.” Central lines are eligible for CLABSI events if a central line has been in place for more than two consecutive calendar days following the first access of the central line during the current admission. Such lines are eligible for CLABSI events and remain eligible for CLABSI events until the day after removal from the body or patient discharge, whichever comes first”[12].

A *laboratory-confirmed bloodstream infection* is classified as neonatal CLABSI if it meets one of the following criteria.

1. The patient has a recognized bacterial or fungal pathogen which is not labelled as a ‘common commensal’ (according to the NHSN Master Organism List [12]) AND is identified from one or more blood specimens obtained by a culture AND is not related to an infection at another site.
2. The patient has clinical symptoms of sepsis according to the treating physician AND organism(s) identified in blood are not related to an infection at another site AND
  - the same common commensal (according to the NHSN Master Organism List [12]) is identified by a culture from two or more blood specimens collected on separate occasions. These separate occasions are defined as at least two separate blood draws collected on the same or consecutive calendar day.
  - OR a common commensal is identified by a culture from only one blood specimen AND C-reactive protein is above 10 mg/L in the first 36 h following the blood draw of the specimen from which the common commensal is identified.

Clinical symptoms must occur within an “infection window” of three days before and/or three days after blood sampling which is in concordance with the CDC guidelines on surveillance of hospital acquired infections [12]. Blood specimens for blood culture can be collected through peripheral venepuncture or can be sampled from central lines and should be obtained in compliance with existing guidelines before the start of antibiotic treatment and under optimal hygienic conditions. CLABSI rates are expressed as the number of CLABSI per 1000 central line-days. Central line-days are counted in calendar days by the number of patients with one or more central line(s) in place during a certain period of time. Only one central line-day per patient is counted per calendar day regardless of the number of central lines present.

## Discussion

Our aim is to achieve nationwide surveillance of CLABSI incidence rates in neonatal intensive care centres in the Netherlands to facilitate intra- and interfacility rate comparisons. Since various methods were used for local monitoring of neonatal CLABSI incidence rates, consensus on CLABSI criteria for standardised surveillance was pivotal. Criteria established by the CDC, ECDC and the NEO-KISS network were not suitable as they do not conform to clinical practice in neonatal care in the Netherlands and presumably many other countries as well. Therefore, new and concise neonatal CLABSI SC were needed that were objective, specified to the neonatal care setting, easy to apply and sustainable. Our consensus procedure successfully resulted in the development of nationwide neonatal CLABSI SC in the Netherlands supported by all NICUs. These criteria are specified to the neonatal population and based on a single blood culture policy for sepsis confirmation.

As recommended by the World Health Organisation (WHO), facility-based CLABSI surveillance should be performed to guide interventions for infection prevention and control [21]. Setting up any surveillance starts with a clear and suitable surveillance case definition. Consensus on neonatal CLABSI criteria is therefore an essential step. Surveillance data are more likely to be accepted when the surveillance case definitions match with the clinicians’ expectations [22, 23]. Therefore, an expert panel composed of clinicians representing the NICUs in the Netherlands participated in a modified Delphi procedure concerning the development of neonatal CLABSI SC.

The expert panel concluded that the internationally used neonatal CLABSI SC (CDC, ECDC and NEO-KISS) were not suitable for the Dutch neonatal setting. The CDC and ECDC criteria would substantially underestimate our neonatal CLABSI incidence. Particularly, CLABSI caused by common commensals (mainly CoNS) are missed since according to these criteria, a second blood culture is required for confirmation while the Netherlands follows a single blood culture policy. CoNS are common CLABSI causing pathogens in neonates, and not including CoNS would result in an underestimation of the true CLABSI incidence rate [24]. A single blood culture policy is preferred due to restricted vessel access in neonates, the potential risk for increased transfusion requirements by repeated blood sampling, the possible rapid deterioration of neonates in the setting of sepsis and the aim to start antibiotics as soon as possible. A large variation in blood culture practices for the diagnosis of bloodstream infections in newborns exists worldwide, ranging from sample site (peripheral, central or both) to the number of blood samples taken [25, 26]. Studies in the neonatal population have shown conflicting results with regard to the need for blood cultures collected from multiple sites for optimal organism detection [27, 28]. In neonatal practices aiming for more than one blood culture for bloodstream infection confirmation, many factors such as technical difficulties can still result in only a single obtained blood specimen [28]. In our opinion, this variation in clinical practice should be taken into account when developing neonatal CLABSI SC in order to make them generally applicable in neonatal care. Our expert panel included C-reactive protein (CRP) as a laboratory marker for CLABSI confirmation in the case of a single CoNS positive blood culture. The value of CRP in diagnosing sepsis in neonates has been debated and previous studies have indicated that neonates with culture-proven bloodstream infection can have low levels of CRP [29–31]. Nevertheless, CRP is still the most commonly used inflammatory marker for this purpose in neonatal care internationally [30]. Panel consensus on CRP was also achieved because its use is in line with a recent introduced national guideline concerning prevention and diagnosis of early onset neonatal sepsis (EONS)[32]. Future research results on the diagnostic accuracy of CRP and possible other inflammatory markers (like procalcitonin) might lead to necessary modifications of the newly developed Dutch neonatal CLABSI criteria.

The German NEO-KISS criteria include alternative laboratory elements (leukocyte and thrombocyte count among others) in addition to CRP for confirmation of CoNS CLABSI. These criteria were assessed as not feasible for surveillance of CLABSI in Dutch neonatal care since they require two out of 16 clinical signs or symptoms. Our expert panel aimed to develop concise neonatal CLABSI SC and aspired to include as few as possible (subjective) elements. Restriction in the elements used in SC is consistent with the increasing use of (semi)automated surveillance methods [33]. The inclusion of numerous (clinical) elements in criteria increases labour intensity when using manual surveillance methods and will complicate the application of semi(automated) surveillance methods linked to electronic patient and laboratory data [16, 33]. With the aim to implement (semi)automated surveillance methods in the future, the expert panel intended to develop sustainable and future proof neonatal CLABSI SC. Limitation of the newly developed Dutch neonatal CLABSI SC for this purpose is the inclusion of “clinical symptoms of sepsis according to treating physician” as a pragmatic element to meet neonatal CLABSI caused by CoNS. Embedding this clinical judgement in (semi)automatic methods might be challenging. Furthermore, there is a risk of overestimating the neonatal CLABSI incidence when using one blood culture in the confirmation of CoNS CLABSI, since contamination of (peripheral) obtained blood samples in newborns is a known possibility [34]. Therefore, the practical application of the Dutch neonatal CLABSI SC and related surveillance outcome data will be regularly evaluated by the Working Group on Neonatal Infectious Diseases of The Section of Neonatology of the Dutch Paediatric Society. However, this potential overestimation would still be consistent and therefore will not interfere with the main purpose of CLABSI surveillance, namely to monitor trends and evaluate the impact of preventive interventions.

Another subject of debate by the expert panel was the included element “organism(s) identified by blood culture should not be related to an infection at another site”. Detection of an infection at another site can be complicated by a possible association between bloodstream infections and peripheral venous catheters (PVCs) in neonates. In intensive care treatment of neonates, PVCs are frequently used in addition to and concurrent with central lines. Furthermore, gram-negative bloodstream infections in preterm neonates specifically (with or without an indwelling central line) can be caused by translocation of bacteria from the gastro-intestinal tract. These factors might therefore lead to an overestimation of the true CLABSI incidence when using the Dutch neonatal CLABSI SC.

When extending national neonatal CLABSI surveillance to enable international comparisons of neonatal CLABSI incidence rates, there is a need for international agreement upon core elements in case definitions. Variations in neonatal clinical practice, such as blood culture policy and the use of inflammatory markers in diagnosing sepsis, require international CLABSI SC that are widely applicable. Slight differences in the surveillance methods used or in the application of neonatal CLABSI definitions are likely to lead to considerable differences in incidence numbers [10]. In anticipation of international consensus on neonatal CLABSI SC, a detailed description of the included elements in research and surveillance CLABSI case definition(s) is crucial for critical interpretation and comparison of neonatal CLABSI incidence rates. When adopting available neonatal CLABSI SC, it is necessary to assess the suitability of the elements used to increase the validity of the incidence data. Future development of criteria for other neonatal HAI is desired and adaptation of available SC to (semi)automatic surveillance methods might be necessary. Generating reliable and valid surveillance data for incidence trend measurement over time is critical to consequently evaluate infection prevention and control interventions. Our standardised consensus procedure can serve as a framework for the development of other HAI SC specified to the neonatal population, both nationally and internationally.

## Conclusions

Monitoring of neonatal CLABSI incidence data is essential for evaluating the impact of preventive measures aiming to improve daily practice. The use of different CLABSI surveillance definitions (inter)nationally impairs accurate interfacility comparison. To obtain standardised nationwide surveillance of neonatal CLABSI rates, a modified Delphi consensus procedure was executed in the Netherlands. The newly developed neonatal CLABSI surveillance criteria are concise, specified to the neonatal population and comply with a single blood culture policy in neonatal clinical practice. International agreement upon neonatal CLABSI criteria is needed to improve the interfacility rate comparison which facilitates best practice identification.

## Data Availability

Data sharing is not applicable to this article as no datasets were generated or analysed during the current study.

## Abbreviations

CDC: Centers for Disease Control and Prevention
CLABSI: Central line-associated bloodstream infections
CoNS: Coagulase-negative Staphylococci species
CRP: C-reactive protein
ECDC: European Centre for Disease Control and Prevention
EONS: Early onset neonatal sepsis
HAI: Hospital-acquired infections
NEO-KISS: NEO-Krankenhaus Infektions Surveillance System
NHSN: National Healthcare Surveillance Network
NICUs: Neonatal intensive care units
PVCs: Peripheral venous catheters
SC: Surveillance criteria
WHO: World Health Organisation
